# 3,5-Di-*O*-benzoyl-1,2-*O*-isopropyl­idene-α-d-*ribo*-hexos-3-ulo-1,4:3,6-difuran­ose

**DOI:** 10.1107/S1600536811024317

**Published:** 2011-06-25

**Authors:** Qiurong Zhang, Xuebin Chen, Nan Zhu, Tengfei Jiang, Hongmin Liu

**Affiliations:** a New Drug Reseach & Development Center, Zhengzhou Univresity, Zhengzhou 450001, People’s Republic of China

## Abstract

The title compound, C_23_H_22_O_8_, is a binary benzoyl ester whose nucleus consists of a fused system made up of a methyl­enedi­oxy ring and two tetra­hydro­furan rings. One of the benzoyl ester groups is attached at the junction of the two tetra­hydro­furan rings. The other is attached to the outer tetra­furan ring. Both the benzoyl ester groups are in an axial conformation with respect to the outer tetrhydro­furan ring. In the crystal, mol­ecules are linked by two weak C—H⋯O hydrogen bonds, forming a chain running parallel to the *a* axis.

## Related literature

For details of the synthesis and absolute configuration of the nucleus, see: Tronchet & Bourgeois (1971 ▶). For applications of the nucleus, see: Xavier *et al.* (2009 ▶); Rajwanshi *et al.* (1999 ▶). For structure of a bicyclo-glycosyl compound, see: Zhang *et al.* (2011 ▶).
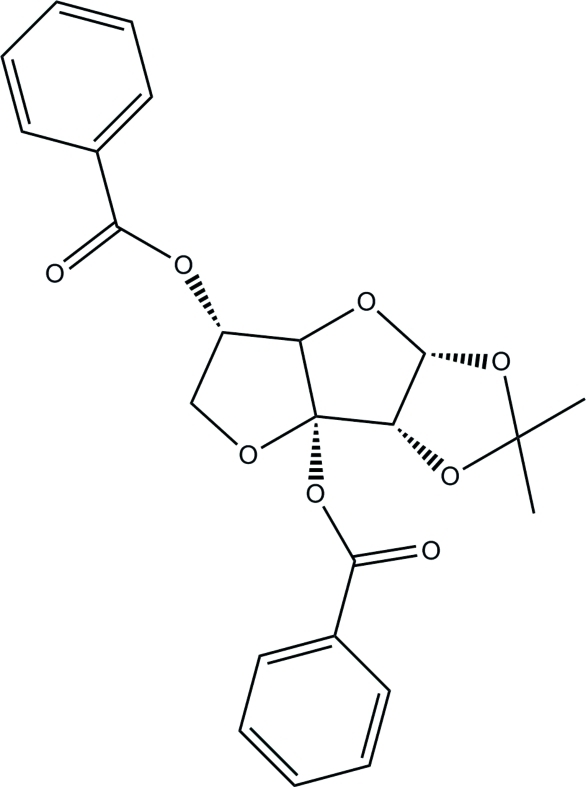


## Experimental

### 

#### Crystal data

C_23_H_22_O_8_
*M* *_r_* = 426.41
Orthorhombic, 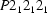

*a* = 6.05837 (10) Å
*b* = 8.33827 (14) Å
*c* = 40.9992 (7) Å
*V* = 2071.13 (6) Å^3^
*Z* = 4
Cu *K*α radiation
μ = 0.87 mm^−1^
*T* = 291 K
0.30 × 0.30 × 0.25 mm

#### Data collection

Agilent Xcalibur Eos Gemini diffractometer
Absorption correction: multi-scan (*CrysAlis PRO*; Agilent, 2011 ▶) *T*_min_ = 0.780, *T*_max_ = 0.812
10556 measured reflections
2421 independent reflections
2335 reflections with *I* > 2σ(*I*)
*R*_int_ = 0.026

#### Refinement

*R*[*F* ^2^ > 2σ(*F* ^2^)] = 0.036
*wR*(*F* ^2^) = 0.094
*S* = 1.06
2421 reflections
282 parameters
H-atom parameters constrained
Δρ_max_ = 0.19 e Å^−3^
Δρ_min_ = −0.26 e Å^−3^

### 

Data collection: *CrysAlis PRO* (Agilent, 2011 ▶); cell refinement: *CrysAlis PRO*; data reduction: *CrysAlis PRO*; program(s) used to solve structure: *SHELXS97* (Sheldrick, 2008 ▶); program(s) used to refine structure: *SHELXL97* (Sheldrick, 2008 ▶); molecular graphics: *OLEX2* (Dolomanov *et al.*, 2009 ▶) and *PLATON* (Spek, 2009 ▶); software used to prepare material for publication: *OLEX2*.

## Supplementary Material

Crystal structure: contains datablock(s) I, global. DOI: 10.1107/S1600536811024317/lw2067sup1.cif

Structure factors: contains datablock(s) I. DOI: 10.1107/S1600536811024317/lw2067Isup2.hkl

Additional supplementary materials:  crystallographic information; 3D view; checkCIF report

## Figures and Tables

**Table 1:** Hydrogen-bond geometry (Å, °)

*D*—H⋯*A*	*D*—H	H⋯*A*	*D*⋯*A*	*D*—H⋯*A*
C2—H2⋯O6^i^	0.98	2.55	3.2793 (17)	131
C4—H4⋯O8^ii^	0.98	2.59	3.4882 (16)	153

Symmetry codes: (i) 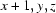; (ii) 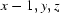.

**Fig. 1.**
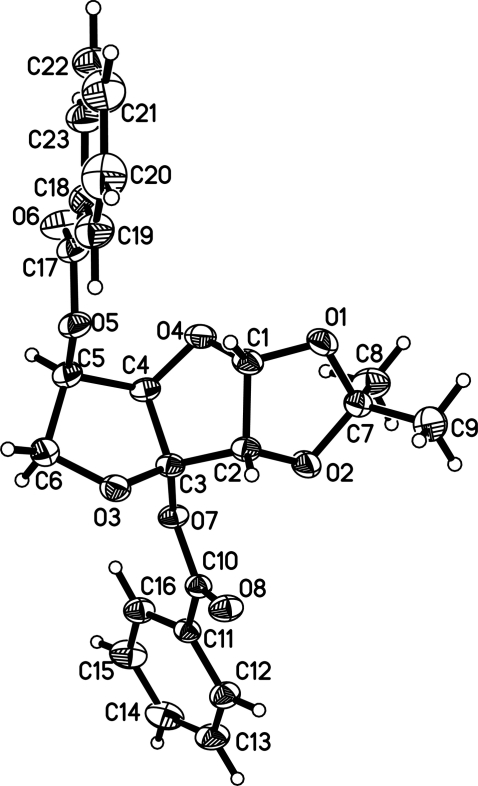
The molecular structure of (I) showing the atomic numbering and 30% probability displacement ellipsoids. H atoms omitted clarity.

**Fig. 2.**
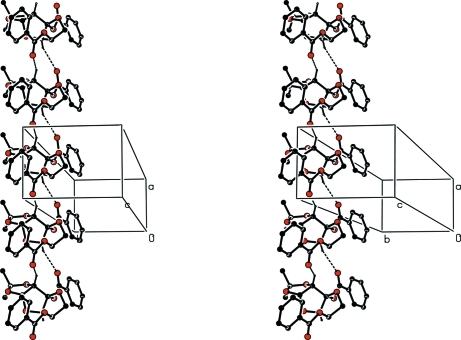
Stereoview of the chain formed by C-H···O hydrogen bonds. Hydrogen atoms not involved in the motifs are not included.

## supplementary crystallographic information

### Comment

C_23_H_22_O_8_, (I), is an important intermediate in the synthesis of bicyclo-glycosyls (Zhang *et al.*, 2011), whose nucleoside derivatives play a vital role as anti-tumour and antivirus agents have been synthesised (Xavier *et al.* 2009; Rajwanshi *et al.* 1999).

The nucleus of molecule (I),Figure 1, consists of three fused rings, a methylenedioxy ring which is linked to two fused tetrahydrofuran rings. The methylenedioxy ring and a tetrahydrofuran ring are oriented in opposite directions with respect to the central tetrahydrofuran. One of the benzoyl ester groups is attached at the junction of the two tetrahydrofuran rings. The other is attached to the outer tetrafuran ring. Both the benzoyl ester groups are in an axial conformation with respect to the outer tetrhydrofuran ring.

The molecules are linked by two weak C-H···O hydrogen bonds, Table 1, to form a chain which runs parallel to the a-axis, Figure 2.

### Experimental

The title compound (I) was synthesized from 1,2;5,6-di-*O*-isopropylidene-α- D-*ribo*-hexofuranosid-3-ulose as described previously by Tronchet, (Tronchet & Bourgeois, 1971), whose starting material was D-glucose. A solution of 1,2;5,6-di-*O*-isopropylidene-α- D-*ribo*-hexofuranosid-3-ulose (1.01 g, 3.87 mmol) in aq. AcOH (60%,15 ml) was stirred at room temperature overnight. The solvent was co-evaporated with toluene and the residue was purified by column chromatography (EtOAc) to pruduce the difuranose compound 1,2-*O*-Isopropylidene-α-D-*ribo*-hexos-3-ulo-1,4:3,6-difuranose(0.79 g). Benzoyl chloride (0.05 ml,9 mmol) was then added to a solution of this difuranose compound in dry pyridine (3 ml). This solution was stirred at room temperature for 2 h. Water (10 ml) was added to the solution, and the mixture was extracted with EtOAc. The combined organic layers were washed with water and dried with anhydrous Na_2_SO_4_. After filtration and evaporation of the solvent, the residue was recrystallised in EtOAc to obtain the title compound as white solid. Crystals suitable for X-ray analysis were grown by slow evaporation from acetone at room temperature for two weeks. mp: 459-460K; *R*_f_ = 0.35 (petroleum ether/EtOAc, 5:1); ^1^H NMR (400 MHz, CDCl_3_) σ: 8.10(4*H*, m), 7.62(m, 2H), 7.49(m, 4H), 6.06(1*H*, d, *J*=3.8 Hz), 5.68(1*H*, dd, *J*=6.1 Hz), 5.23(1*H*, d, *J*=3.8 Hz), 5.12(1*H*, d, *J*=4.7,6.0 Hz), 4.58(1*H*, dd, *J*=9.4,7.0 Hz), 4.27(1*H*, dd, *J*=9.4,6.0 Hz), 1.52(3*H*, s), 1.37 (3*H*, s). ^13^C NMR (100 MHz, CDCl_3_) σ: 165.9, 164.4, 133.6, 133.5, 130.0, 129.9, 129.5, 129.1, 128.5, 128.5, 113.8, 113.2, 107.4, 82.9, 81.8, 72.6, 72.2, 27.2, 27.2.

### Refinement

All H atoms were placed geometrically and treated as riding on their parent atoms with C—H are 0.93Å(aromatic), 0.96Å(methyl), 0.97Å(methylene) and 0.98Å(aliphatic) with *U*_iso_(H) =1.2*U*_eq_(C).

In the absence of any significant anomalous scatterers in the molecule, attempts to confirm the absolute structure by refinement of the Flack parameter in the presence of 1675 sets of Friedel equivalents led to an inconclusive value of -0.10 (14). Therefore, the Friedel pairs were merged before the final refinement and the absolute configuration was assigned to correspond with that of the known chiral centres in a precursor molecule, which remained unchanged during the synthesis of the title compound.

### Crystal data

**Table:**

C_23_H_22_O_8_	*D*_x_ = 1.367 Mg m^−^^3^
*M**_r_* = 426.41	Melting point = 459–460 K
Orthorhombic, *P*2_1_2_1_2_1_	Cu *K*α radiation, λ = 1.5418 Å
*a* = 6.05837 (10) Å	Cell parameters from 5890 reflections
*b* = 8.33827 (14) Å	θ = 3.2–72.3°
*c* = 40.9992 (7) Å	µ = 0.87 mm^−^^1^
*V* = 2071.13 (6) Å^3^	*T* = 291 K
*Z* = 4	Prism, colourless
*F*(000) = 896	0.30 × 0.30 × 0.25 mm

### Data collection

**Table:**

Agilent Xcalibur Eos Gemini diffractometer	2421 independent reflections
Radiation source: Enhance (Cu) X-ray Source	2335 reflections with *I* > 2σ(*I*)
graphite	*R*_int_ = 0.026
Detector resolution: 16.2312 pixels mm^-1^	θ_max_ = 72.3°, θ_min_ = 4.3°
ω scans	*h* = −7→7
Absorption correction: multi-scan (*CrysAlis PRO*; Agilent, 2011)	*k* = −9→10
*T*_min_ = 0.780, *T*_max_ = 0.812	*l* = −21→50
10556 measured reflections	

### Refinement

**Table:**

Refinement on *F*^2^	Primary atom site location: structure-invariant direct methods
Least-squares matrix: full	Secondary atom site location: difference Fourier map
*R*[*F*^2^ > 2σ(*F*^2^)] = 0.036	Hydrogen site location: inferred from neighbouring sites
*wR*(*F*^2^) = 0.094	H-atom parameters constrained
*S* = 1.06	*w* = 1/[σ^2^(*F*_o_^2^) + (0.0643*P*)^2^ + 0.1182*P*] where *P* = (*F*_o_^2^ + 2*F*_c_^2^)/3
2421 reflections	(Δ/σ)_max_ = 0.001
282 parameters	Δρ_max_ = 0.19 e Å^−^^3^
0 restraints	Δρ_min_ = −0.26 e Å^−^^3^

### Special details

**Table:**

Geometry. All esds (except the esd in the dihedral angle between two l.s. planes) are estimated using the full covariance matrix. The cell esds are taken into account individually in the estimation of esds in distances, angles and torsion angles; correlations between esds in cell parameters are only used when they are defined by crystal symmetry. An approximate (isotropic) treatment of cell esds is used for estimating esds involving l.s. planes.
Refinement. Refinement of *F*^2^ against ALL reflections. The weighted *R*-factor *wR* and goodness of fit *S* are based on *F*^2^, conventional *R*-factors *R* are based on *F*, with *F* set to zero for negative *F*^2^. The threshold expression of *F*^2^ > σ(*F*^2^) is used only for calculating *R*-factors(gt) *etc*. and is not relevant to the choice of reflections for refinement. *R*-factors based on *F*^2^ are statistically about twice as large as those based on *F*, and *R*- factors based on ALL data will be even larger.

### Fractional atomic coordinates and isotropic or equivalent isotropic displacement parameters (Å^2^)

**Table:**

	*x*	*y*	*z*	*U*_iso_*/*U*_eq_	
O1	0.7379 (3)	1.23548 (17)	0.88731 (3)	0.0556 (4)	
O2	0.8454 (3)	1.07286 (15)	0.84526 (3)	0.0553 (4)	
O3	0.7285 (2)	0.70109 (16)	0.88507 (3)	0.0458 (3)	
O4	0.4603 (2)	1.04625 (17)	0.88971 (3)	0.0460 (3)	
O5	0.4346 (2)	0.82295 (17)	0.93608 (3)	0.0462 (3)	
O6	0.1023 (3)	0.9393 (3)	0.94092 (4)	0.0694 (5)	
O7	0.6458 (2)	0.78758 (16)	0.83253 (3)	0.0396 (3)	
O8	1.0139 (2)	0.75343 (19)	0.82694 (3)	0.0477 (3)	
C1	0.6881 (4)	1.0796 (2)	0.89615 (4)	0.0436 (4)	
H1	0.7261	1.0586	0.9190	0.052*	
C2	0.8210 (3)	0.9737 (2)	0.87294 (4)	0.0388 (3)	
H2	0.9629	0.9401	0.8822	0.047*	
C3	0.6676 (3)	0.8329 (2)	0.86608 (4)	0.0359 (3)	
C4	0.4365 (3)	0.8886 (2)	0.87778 (4)	0.0395 (4)	
H4	0.3302	0.8855	0.8598	0.047*	
C5	0.3748 (4)	0.7633 (2)	0.90435 (4)	0.0469 (4)	
H5	0.2192	0.7316	0.9032	0.056*	
C6	0.5277 (4)	0.6267 (2)	0.89661 (5)	0.0576 (6)	
H6B	0.5562	0.5628	0.9159	0.069*	
H6A	0.4647	0.5581	0.8799	0.069*	
C7	0.8226 (4)	1.2368 (2)	0.85457 (4)	0.0457 (4)	
C8	0.6599 (5)	1.3136 (4)	0.83165 (6)	0.0666 (6)	
H8C	0.6363	1.4231	0.8380	0.100*	
H8B	0.5225	1.2564	0.8325	0.100*	
H8A	0.7170	1.3102	0.8098	0.100*	
C9	1.0429 (4)	1.3199 (3)	0.85511 (7)	0.0701 (6)	
H9B	1.1420	1.2623	0.8691	0.105*	
H9C	1.0248	1.4273	0.8631	0.105*	
H9A	1.1025	1.3232	0.8334	0.105*	
C10	0.8314 (3)	0.7426 (2)	0.81616 (4)	0.0361 (3)	
C11	0.7740 (3)	0.6775 (2)	0.78333 (4)	0.0369 (3)	
C12	0.9415 (4)	0.6641 (3)	0.76038 (4)	0.0491 (4)	
H12	1.0828	0.7009	0.7652	0.059*	
C13	0.8968 (4)	0.5954 (3)	0.73032 (5)	0.0582 (5)	
H13	1.0080	0.5875	0.7148	0.070*	
C14	0.6894 (5)	0.5390 (3)	0.72342 (5)	0.0623 (6)	
H14	0.6609	0.4922	0.7033	0.075*	
C15	0.5228 (4)	0.5511 (3)	0.74619 (6)	0.0621 (6)	
H15	0.3831	0.5112	0.7415	0.075*	
C16	0.5637 (3)	0.6229 (3)	0.77623 (5)	0.0479 (4)	
H16	0.4507	0.6341	0.7914	0.057*	
C17	0.2826 (3)	0.9126 (3)	0.95157 (4)	0.0477 (4)	
C18	0.3653 (4)	0.9719 (2)	0.98356 (4)	0.0475 (4)	
C19	0.5728 (5)	0.9336 (3)	0.99530 (5)	0.0605 (5)	
H19	0.6685	0.8707	0.9830	0.073*	
C20	0.6362 (6)	0.9909 (4)	1.02587 (6)	0.0791 (8)	
H20	0.7746	0.9653	1.0342	0.095*	
C21	0.4942 (7)	1.0852 (4)	1.04379 (6)	0.0831 (9)	
H21	0.5379	1.1235	1.0641	0.100*	
C22	0.2893 (6)	1.1233 (4)	1.03201 (6)	0.0788 (8)	
H22	0.1942	1.1865	1.0443	0.095*	
C23	0.2242 (5)	1.0674 (3)	1.00179 (5)	0.0606 (6)	
H23	0.0856	1.0939	0.9936	0.073*	

### Atomic displacement parameters (Å^2^)

**Table:**

	*U*^11^	*U*^22^	*U*^33^	*U*^12^	*U*^13^	*U*^23^
O1	0.0844 (11)	0.0392 (6)	0.0433 (7)	−0.0017 (7)	0.0146 (7)	−0.0098 (5)
O2	0.0874 (11)	0.0392 (6)	0.0392 (6)	−0.0010 (7)	0.0204 (7)	−0.0044 (5)
O3	0.0562 (8)	0.0416 (6)	0.0396 (6)	0.0110 (6)	0.0062 (6)	0.0031 (5)
O4	0.0521 (7)	0.0434 (6)	0.0424 (6)	0.0123 (6)	0.0078 (6)	−0.0030 (5)
O5	0.0523 (7)	0.0552 (7)	0.0311 (5)	0.0076 (6)	0.0072 (5)	−0.0005 (5)
O6	0.0528 (8)	0.1047 (14)	0.0508 (8)	0.0200 (10)	−0.0035 (7)	−0.0178 (9)
O7	0.0390 (6)	0.0500 (6)	0.0298 (5)	0.0027 (5)	0.0007 (4)	−0.0102 (5)
O8	0.0401 (6)	0.0607 (8)	0.0424 (6)	0.0043 (6)	−0.0024 (5)	−0.0143 (6)
C1	0.0598 (10)	0.0410 (8)	0.0299 (7)	0.0052 (8)	0.0020 (8)	−0.0058 (7)
C2	0.0439 (8)	0.0417 (8)	0.0307 (7)	0.0037 (7)	0.0010 (7)	−0.0056 (6)
C3	0.0413 (8)	0.0388 (8)	0.0275 (7)	0.0060 (7)	−0.0009 (6)	−0.0032 (6)
C4	0.0408 (8)	0.0491 (9)	0.0286 (7)	0.0049 (8)	0.0027 (6)	−0.0056 (7)
C5	0.0541 (10)	0.0506 (9)	0.0360 (8)	−0.0012 (9)	0.0097 (7)	−0.0050 (7)
C6	0.0780 (15)	0.0425 (9)	0.0523 (10)	0.0011 (10)	0.0217 (11)	−0.0001 (8)
C7	0.0545 (10)	0.0393 (8)	0.0435 (9)	0.0008 (9)	0.0076 (8)	−0.0036 (7)
C8	0.0701 (15)	0.0739 (14)	0.0559 (12)	0.0172 (13)	−0.0006 (12)	−0.0003 (11)
C9	0.0634 (14)	0.0671 (14)	0.0800 (15)	−0.0100 (13)	0.0069 (12)	−0.0134 (13)
C10	0.0408 (8)	0.0361 (7)	0.0315 (7)	0.0021 (7)	0.0025 (7)	−0.0039 (6)
C11	0.0454 (9)	0.0364 (7)	0.0290 (6)	0.0051 (7)	0.0004 (6)	−0.0020 (6)
C12	0.0515 (10)	0.0567 (10)	0.0391 (8)	0.0000 (9)	0.0066 (8)	−0.0065 (8)
C13	0.0734 (14)	0.0667 (12)	0.0346 (8)	0.0088 (12)	0.0122 (9)	−0.0097 (9)
C14	0.0805 (15)	0.0693 (13)	0.0370 (8)	0.0155 (13)	−0.0122 (10)	−0.0208 (9)
C15	0.0572 (12)	0.0734 (14)	0.0558 (11)	0.0064 (12)	−0.0127 (10)	−0.0247 (11)
C16	0.0473 (10)	0.0549 (10)	0.0414 (8)	0.0027 (9)	−0.0010 (8)	−0.0117 (8)
C17	0.0512 (11)	0.0564 (10)	0.0355 (8)	0.0069 (9)	0.0068 (8)	0.0003 (8)
C18	0.0607 (11)	0.0502 (9)	0.0315 (7)	0.0008 (9)	0.0058 (8)	0.0047 (7)
C19	0.0694 (13)	0.0703 (13)	0.0420 (9)	0.0067 (13)	−0.0031 (10)	0.0033 (9)
C20	0.091 (2)	0.0973 (19)	0.0493 (11)	−0.0043 (17)	−0.0210 (13)	0.0079 (13)
C21	0.123 (3)	0.0871 (18)	0.0391 (10)	−0.017 (2)	−0.0067 (14)	−0.0085 (12)
C22	0.114 (2)	0.0735 (15)	0.0488 (11)	−0.0001 (17)	0.0131 (15)	−0.0166 (12)
C23	0.0725 (14)	0.0620 (12)	0.0473 (10)	0.0087 (12)	0.0082 (10)	−0.0061 (9)

### Geometric parameters (Å, °)

**Table:**

O1—C1	1.382 (2)	C8—H8A	0.9600
O1—C7	1.437 (2)	C9—H9B	0.9600
O2—C2	1.412 (2)	C9—H9C	0.9600
O2—C7	1.426 (2)	C9—H9A	0.9600
O3—C3	1.397 (2)	C10—C11	1.492 (2)
O3—C6	1.445 (3)	C11—C16	1.384 (3)
O4—C4	1.410 (2)	C11—C12	1.389 (3)
O4—C1	1.432 (3)	C12—C13	1.386 (3)
O5—C17	1.346 (2)	C12—H12	0.9300
O5—C5	1.439 (2)	C13—C14	1.371 (4)
O6—C17	1.197 (3)	C13—H13	0.9300
O7—C10	1.362 (2)	C14—C15	1.378 (4)
O7—C3	1.4328 (17)	C14—H14	0.9300
O8—C10	1.194 (2)	C15—C16	1.392 (3)
C1—C2	1.528 (2)	C15—H15	0.9300
C1—H1	0.9800	C16—H16	0.9300
C2—C3	1.523 (3)	C17—C18	1.489 (3)
C2—H2	0.9800	C18—C19	1.383 (3)
C3—C4	1.551 (2)	C18—C23	1.387 (3)
C4—C5	1.555 (3)	C19—C20	1.395 (3)
C4—H4	0.9800	C19—H19	0.9300
C5—C6	1.502 (3)	C20—C21	1.378 (5)
C5—H5	0.9800	C20—H20	0.9300
C6—H6B	0.9700	C21—C22	1.369 (5)
C6—H6A	0.9700	C21—H21	0.9300
C7—C9	1.503 (3)	C22—C23	1.381 (3)
C7—C8	1.505 (3)	C22—H22	0.9300
C8—H8C	0.9600	C23—H23	0.9300
C8—H8B	0.9600		
			
C1—O1—C7	109.27 (13)	C7—C8—H8A	109.5
C2—O2—C7	109.65 (13)	H8C—C8—H8A	109.5
C3—O3—C6	107.35 (16)	H8B—C8—H8A	109.5
C4—O4—C1	110.09 (14)	C7—C9—H9B	109.5
C17—O5—C5	116.50 (16)	C7—C9—H9C	109.5
C10—O7—C3	118.02 (13)	H9B—C9—H9C	109.5
O1—C1—O4	110.15 (17)	C7—C9—H9A	109.5
O1—C1—C2	105.39 (15)	H9B—C9—H9A	109.5
O4—C1—C2	106.30 (14)	H9C—C9—H9A	109.5
O1—C1—H1	111.6	O8—C10—O7	124.12 (14)
O4—C1—H1	111.6	O8—C10—C11	125.24 (15)
C2—C1—H1	111.6	O7—C10—C11	110.64 (14)
O2—C2—C3	111.52 (14)	C16—C11—C12	120.26 (16)
O2—C2—C1	102.53 (14)	C16—C11—C10	121.59 (15)
C3—C2—C1	103.82 (16)	C12—C11—C10	118.05 (17)
O2—C2—H2	112.7	C13—C12—C11	119.6 (2)
C3—C2—H2	112.7	C13—C12—H12	120.2
C1—C2—H2	112.7	C11—C12—H12	120.2
O3—C3—O7	110.60 (13)	C14—C13—C12	120.3 (2)
O3—C3—C2	110.03 (14)	C14—C13—H13	119.9
O7—C3—C2	115.89 (13)	C12—C13—H13	119.9
O3—C3—C4	107.55 (14)	C13—C14—C15	120.39 (18)
O7—C3—C4	107.02 (13)	C13—C14—H14	119.8
C2—C3—C4	105.26 (13)	C15—C14—H14	119.8
O4—C4—C3	107.09 (15)	C14—C15—C16	120.0 (2)
O4—C4—C5	114.09 (13)	C14—C15—H15	120.0
C3—C4—C5	103.45 (14)	C16—C15—H15	120.0
O4—C4—H4	110.6	C11—C16—C15	119.44 (19)
C3—C4—H4	110.6	C11—C16—H16	120.3
C5—C4—H4	110.6	C15—C16—H16	120.3
O5—C5—C6	107.33 (18)	O6—C17—O5	123.76 (18)
O5—C5—C4	109.90 (15)	O6—C17—C18	124.54 (19)
C6—C5—C4	102.30 (15)	O5—C17—C18	111.69 (17)
O5—C5—H5	112.3	C19—C18—C23	120.3 (2)
C6—C5—H5	112.3	C19—C18—C17	122.40 (19)
C4—C5—H5	112.3	C23—C18—C17	117.3 (2)
O3—C6—C5	105.23 (16)	C18—C19—C20	118.9 (3)
O3—C6—H6B	110.7	C18—C19—H19	120.5
C5—C6—H6B	110.7	C20—C19—H19	120.5
O3—C6—H6A	110.7	C21—C20—C19	120.2 (3)
C5—C6—H6A	110.7	C21—C20—H20	119.9
H6B—C6—H6A	108.8	C19—C20—H20	119.9
O2—C7—O1	106.09 (14)	C22—C21—C20	120.7 (2)
O2—C7—C9	111.09 (19)	C22—C21—H21	119.7
O1—C7—C9	107.87 (18)	C20—C21—H21	119.7
O2—C7—C8	107.70 (18)	C21—C22—C23	119.8 (3)
O1—C7—C8	110.62 (18)	C21—C22—H22	120.1
C9—C7—C8	113.3 (2)	C23—C22—H22	120.1
C7—C8—H8C	109.5	C22—C23—C18	120.1 (3)
C7—C8—H8B	109.5	C22—C23—H23	120.0
H8C—C8—H8B	109.5	C18—C23—H23	120.0
			
C7—O1—C1—O4	−93.88 (18)	O5—C5—C6—O3	−81.08 (19)
C7—O1—C1—C2	20.4 (2)	C4—C5—C6—O3	34.6 (2)
C4—O4—C1—O1	141.46 (13)	C2—O2—C7—O1	−11.8 (2)
C4—O4—C1—C2	27.76 (18)	C2—O2—C7—C9	105.2 (2)
C7—O2—C2—C3	133.74 (17)	C2—O2—C7—C8	−130.29 (19)
C7—O2—C2—C1	23.2 (2)	C1—O1—C7—O2	−6.4 (2)
O1—C1—C2—O2	−26.6 (2)	C1—O1—C7—C9	−125.5 (2)
O4—C1—C2—O2	90.33 (18)	C1—O1—C7—C8	110.2 (2)
O1—C1—C2—C3	−142.79 (15)	C3—O7—C10—O8	−7.7 (3)
O4—C1—C2—C3	−25.87 (18)	C3—O7—C10—C11	171.87 (13)
C6—O3—C3—O7	−91.18 (17)	O8—C10—C11—C16	159.8 (2)
C6—O3—C3—C2	139.50 (15)	O7—C10—C11—C16	−19.8 (2)
C6—O3—C3—C4	25.35 (17)	O8—C10—C11—C12	−16.5 (3)
C10—O7—C3—O3	−66.81 (19)	O7—C10—C11—C12	163.94 (17)
C10—O7—C3—C2	59.3 (2)	C16—C11—C12—C13	−0.1 (3)
C10—O7—C3—C4	176.32 (14)	C10—C11—C12—C13	176.24 (18)
O2—C2—C3—O3	149.85 (14)	C11—C12—C13—C14	−0.9 (3)
C1—C2—C3—O3	−100.44 (16)	C12—C13—C14—C15	0.5 (4)
O2—C2—C3—O7	23.5 (2)	C13—C14—C15—C16	0.9 (4)
C1—C2—C3—O7	133.17 (15)	C12—C11—C16—C15	1.5 (3)
O2—C2—C3—C4	−94.54 (16)	C10—C11—C16—C15	−174.7 (2)
C1—C2—C3—C4	15.16 (17)	C14—C15—C16—C11	−1.9 (4)
C1—O4—C4—C3	−17.61 (17)	C5—O5—C17—O6	2.7 (3)
C1—O4—C4—C5	96.22 (18)	C5—O5—C17—C18	−178.13 (16)
O3—C3—C4—O4	117.80 (14)	O6—C17—C18—C19	177.9 (2)
O7—C3—C4—O4	−123.34 (14)	O5—C17—C18—C19	−1.3 (3)
C2—C3—C4—O4	0.50 (16)	O6—C17—C18—C23	−1.5 (3)
O3—C3—C4—C5	−3.04 (17)	O5—C17—C18—C23	179.36 (19)
O7—C3—C4—C5	115.82 (15)	C23—C18—C19—C20	0.8 (4)
C2—C3—C4—C5	−120.34 (15)	C17—C18—C19—C20	−178.5 (2)
C17—O5—C5—C6	−161.55 (18)	C18—C19—C20—C21	−0.6 (4)
C17—O5—C5—C4	87.9 (2)	C19—C20—C21—C22	0.4 (5)
O4—C4—C5—O5	−21.2 (2)	C20—C21—C22—C23	−0.4 (5)
C3—C4—C5—O5	94.79 (16)	C21—C22—C23—C18	0.6 (4)
O4—C4—C5—C6	−134.95 (18)	C19—C18—C23—C22	−0.8 (4)
C3—C4—C5—C6	−18.98 (19)	C17—C18—C23—C22	178.6 (2)
C3—O3—C6—C5	−38.6 (2)		

### Hydrogen-bond geometry (Å, °)

**Table:**

*D*—H···*A*	*D*—H	H···*A*	*D*···*A*	*D*—H···*A*
C2—H2···O6^i^	0.98	2.55	3.2793 (17)	131
C4—H4···O8^ii^	0.98	2.59	3.4882 (16)	153

Symmetry codes: (i) *x*+1, *y*, *z*; (ii) *x*−1, *y*, *z*.
